# Drinking Patterns and Alcohol-Related Birth Defects

**Published:** 2001

**Authors:** Susan E. Maier, James R. West

**Affiliations:** Susan E. Maier, Ph.D., is a research assistant professor in the Department of Human Anatomy and Medical Neurobiology, College of Medicine, The Texas A&M University System Health Science Center, College Station, Texas. James R. West, Ph.D., is interim vice president for research at The Texas A&M University System Health Science Center, and professor and head of the Department of Human Anatomy and Medical Neurobiology, College of Medicine, The Texas A&M University System Health Science Center, College Station, Texas

**Keywords:** fetal alcohol syndrome, alcohol-related neurodevelopmental disorder, binge AOD (alcohol or other drug) use, AOD use pattern, AOD use frequency, pregnancy, animal model, human study

## Abstract

The consequences of maternal alcohol use during pregnancy on the outcome of offspring depend, among other factors, on the amount and pattern of alcohol consumption. Animal studies found that bingelike drinking patterns, in which the fetus is exposed to high blood alcohol concentrations (BACs) over relatively short periods of time, are particularly harmful, even if the overall alcohol amount consumed is less than those of more continuous drinking patterns. Long-term studies in humans have confirmed that children of binge-drinking mothers exhibited especially severe cognitive and behavioral deficits. Binge drinking may be particularly harmful because it results in high BACs, may occur during critical periods of brain development, and may be associated with repeated withdrawal episodes.

Alcohol abuse during pregnancy is the leading preventable cause of mental retardation in offspring in the United States. The most severe consequences of maternal alcohol abuse are fetal alcohol syndrome (FAS) and alcohol-related neurodevelopmental disorder (ARND) (see Stratton et al. 1996), both of which are associated with substantial cognitive and behavioral deficits. The number of women who engage in heavy alcohol consumption during pregnancy, however, surpasses the total number of children diagnosed with either FAS or ARND, meaning that not every child whose mother drank alcohol during pregnancy develops FAS or ARND. Moreover, the degree to which people with FAS or ARND are impaired differs from person to person. Several factors may contribute to this variation in the consequences of maternal drinking. These factors include, but are not limited to, the following:

- Maternal drinking pattern
- Differences in maternal metabolism
- Differences in genetic susceptibility
- Timing of the alcohol consumption during pregnancy
- Variation in the vulnerability of different brain regions.

All of these risk factors are important when assessing the effects of alcohol exposure on fetal brain development. Nevertheless, those factors that alter the peak blood alcohol concentration (BAC) experienced by the embryo or the fetus (e.g., maternal drinking patterns and maternal metabolism) are most likely to affect the occurrence and severity of alcohol-induced developmental brain injury. This article explores the consequences maternal drinking patterns have on prenatal alcohol exposure. After defining a commonly used measure to record alcohol consumption (i.e., a “standard drink”) and drinking patterns, the article reviews animal studies that have analyzed the effects of different alcohol consumption patterns. The article then discusses evidence from human studies indicating that a certain drinking pattern (i.e., binge drinking) is particularly harmful to the fetus and presents some of the factors that may contribute to binge drinking’s adverse effects.

## Defining Alcohol Consumption and Drinking Patterns

Determining the level of alcohol consumption that is dangerous to a developing embryo or fetus is a complex issue that requires researchers to define the factors influencing peak BACs after any given alcohol consumption. These variables include both the amount consumed and the pattern in which the amount is consumed.

### Determining the Amount of Alcohol Consumed

Historically, part of the problem in determining what level of alcohol consumption during pregnancy is harmful has been related to how to define consumption levels. The conventional method for measuring alcohol consumption in humans has been through interviews in which the participants are asked to recall their alcohol consumption (e.g., the number of drinks consumed) during a specific time period (e.g., a week). Researchers then use the information from those interviews to convert the reported alcohol consumption into quantifiable measures, such as total grams of alcohol or ounces of absolute alcohol consumed per week. To facilitate this conversion and generate a standardized measure for reporting and analyzing alcohol consumption, regardless of the type of alcoholic drink consumed, investigators must define a “standard drink” that contains a specific, standardized amount of absolute alcohol regardless of the type of beverage. A standard drink frequently is defined as 12 fluid ounces of beer, 5 fluid ounces of wine, or 1.5 fluid ounces of hard liquor. Using this measure, investigators can categorize subjects into groups based on their amount of alcohol consumption, which then allows for hypothetical statistical tests relating maternal alcohol consumption to deficits in the offspring.

### Determining Drinking Patterns

Although information about standard drinks is suitable for calculating the amount of alcohol consumed, this knowledge yields limited information about the pattern of alcohol drinking. A drinking pattern is characterized by the amount of alcohol consumed and the time during which it is ingested. For example, a given amount of alcohol may be consumed in a continuous pattern. In that instance, the total amount of alcohol consumed is spread over a long duration (e.g., 1 week or 1 month) and individual drinks typically are spaced consistently (e.g., 2 drinks per week or 1 drink per day). Conversely, the same amount of alcohol may be consumed in a bingelike pattern. This means that total alcohol consumption is compressed into a short time period (e.g., 1 evening or 1 day) and the time between consumption of individual beverages is short and possibly inconsistent (e.g., a steady consumption of beer interspersed with liquor shots) during that binge. Thus, a woman who reports consuming 7 drinks per week may consume all 7 drinks in 1 evening (i.e., binge) or consume 1 drink per day (i.e., not binge).

These differences in drinking patterns can be crucial in determining the effects maternal alcohol consumption during pregnancy have on the offspring. For example, assume that one pregnant woman reports consuming five drinks per week and another woman reports consuming seven drinks per week. At first glance, one would assume that the women consuming five drinks would do less harm to her fetus than the woman consuming seven drinks. Now assume that the first woman consumes all her drinks on one occasion (i.e., exhibits binge drinking), whereas the second woman consumes one drink per day (i.e., exhibits a continuous drinking pattern). In this scenario, the first woman actually exposes herself (and her fetus) to considerably higher peak BACs than the second woman and, thereby, increases the risk of alcohol-related damage to her fetus.

Researchers and clinicians are particularly concerned about women who consume alcohol in a bingelike pattern before they realize that they are pregnant (referred to as pregnancy recognition) as well as during early pregnancy. The reason for this concern involves the relationship between the timing of these bingelike episodes and the timing of critical periods of fetal brain development. Although heavy alcohol consumption throughout pregnancy leads to a significant risk of brain injury to the developing fetus, the fetus is especially vulnerable to alcohol-induced brain injury during specific stages of brain development, many of which occur early during pregnancy (Ikonomidou et al. 2000; West 1987). If a woman binge drinks during such a critical stage, significant harm may be inflicted on the developing fetal brain. The resulting deficits can range from gross structural abnormalities, such as small brain size (i.e., microencephaly) with significantly altered brain circuitry (e.g., shrinkage or even complete absence of the corpus callosum^1^), to the more subtle, but nonetheless significant, loss of specific nerve cells (i.e., neurons) in a particular brain region. Not surprisingly, such alterations in the normal development of the structural and neurochemical composition of the brain can have deleterious behavioral consequences (e.g., learning or attention deficits) that may not manifest until a later age.

To address these issues and more accurately determine pregnant women’s amount and pattern of alcohol consumption, clinical researchers more recently have begun to ask women about the number of drinks consumed per occasion and the maximum number of drinks on any one occasion. This change in focus about how alcohol consumption should be measured and reported reinforces pregnant women’s recognition that binge drinking is a significant risk factor for fetal development.

## Animal Studies of Consequences of Maternal Drinking Patterns

For many reasons, it is neither ethical nor practical to give alcohol to pregnant women in order to evaluate dose-response effects of alcohol exposure on the developing fetus. In contrast, animal studies enable researchers to control fetal alcohol exposure in terms of size, number, pattern, and timing of alcohol doses given to the mother. Thus, researchers can assign experimental animals (e.g., rats, mice, sheep, and nonhuman primates) to particular groups and administer alcohol to them at specific doses (measured in grams per unit of body weight, such as grams per kilogram [g/kg]) and in specific patterns. This practice ensures uniform dosing (i.e., equal amounts and patterns) for all animals included in the research and, therefore, is suitable for examining issues related to the effects of alcohol exposure on the developing fetal brain, including threshold levels and differences between exposure patterns.

### Extrapolating Animal Studies to Humans

Many animal studies have been undertaken using the basic methodology described in the preceding paragraph, and the results have provided a wealth of information about the adverse effects of alcohol exposure on brain development. To what extent, however, are the results of those studies directly comparable to humans and can, therefore, they provide a basis for counseling pregnant women? To be able to draw meaningful conclusions, researchers must choose measures that apply equally to experimental animals and humans. For example, the alcohol dose administered is not a good measure for extrapolating results of animal studies to humans, because alcohol metabolism differs among various species. Consequently, the same alcohol dose in terms of grams of alcohol per unit of body weight may have vastly different consequences for the human fetus than for an animal fetus.

Behavioral expression of alcohol intoxication is an equally unreliable measure, because even among humans, the level of intoxication following a given alcohol dose can differ vastly. For example, it is well known that chronic alcoholics can have BACs well over 200 milligrams per deciliter (mg/dL) (i.e., 0.20 percent^2^) and still appear sober, because they have developed tolerance to alcohol’s effects. In one study, a sample of people who used emergency room services after confirmed alcohol consumption but who appeared sober (i.e., based on four different criteria, these people did not look or act inebriated) actually had BACs averaging 268 mg/dL (Urso et al. 1981). Consequently, the precision of behavioral measures of sobriety or intoxication is questionable, because these measures can be influenced heavily by behavioral tolerance to alcohol’s intoxicating effect.

The most direct method to evaluate the amount of alcohol given to a subject and to compare the effects of different doses or among different individuals is to measure the BAC. The BAC precisely reflects the amount of alcohol that is present in the subject’s bloodstream at a given time after alcohol administration. Although this measure is difficult to glean from human subjects in clinical studies, in which researchers ask women to recall their level of alcohol consumption over a given time period, it is the preferred method when evaluating the effects of alcohol on brain development in experimental animals. Nevertheless, the BAC can be influenced by several factors other than the alcohol dose administered. These factors include the alcohol concentration of the beverage (Maier et al. 1995); concurrent use of other drugs (Chen et al. 1999); gender (Freeza et al. 1990); and the amount of time elapsed between eating and drinking, which influences how quickly the alcohol passes through the stomach and is absorbed in the intestine. Because these factors are similar for both humans and animals, however, the BAC has been adopted as a reliable method to equate alcohol effects between animals and humans and is the measure least open to problems in interpretation.

Another factor to consider when extrapolating results of animal studies to humans are species differences in the timing of various developmental stages. For example, the equivalent of the brain development found in human fetuses during the third trimester occurs in rats after birth, roughly between postnatal days 1 to 12 (Dobbing and Sands 1979). Accordingly, researchers often use newborn rats (i.e., at postnatal days 4 to 10) to examine alcohol’s effects on brain development in order to derive a direct correlation between BAC and subsequent brain injury from individual rats. Nevertheless, researchers have found that both the rat fetus (Maier et al. 1996) and the sheep fetus (Cudd et al. 2001) exhibit the same BACs as the mother during various trimester equivalents. Therefore, the relationship between BAC and brain injury can be determined regardless of the timing of alcohol exposure in relation to the trimesters.

### Results of Animal Studies on the Effects of Maternal Drinking Patterns

Animal models have been used extensively to investigate the relationship among alcohol levels, drinking patterns, and brain damage. Several noteworthy studies have systematically examined these research questions using both rats and nonhuman primates. Some of those studies have demonstrated a clear relationship between the alcohol dose and the severity of gross brain growth restriction. In one study that used artificial rearing methods^3^ (Bonthius and West 1988), groups of newborn rats were exposed daily during the early postnatal period to a wide range of alcohol doses (i.e., 2.5 to 7.5 g/kg/day). The peak BACs ranged from 30 mg/dL to 525 mg/dL^4^ for the various alcohol-treated groups. The investigators found a near-linear inverse relationship between alcohol dose and total brain weight—that is, higher alcohol doses resulted in lower brain weight. Thus, for a given exposure pattern, higher alcohol doses resulted in both higher BACs and more severe injury to the developing brain.

Other findings from the same research group support the hypothesis that binge-like alcohol exposure is more harmful than non-binge exposure. In that study, Pierce and West (1986) compared the effects of equal total amounts of alcohol administered in different exposure patterns on brain growth in rats. Using artificial rearing methods, the researchers exposed groups of neonatal rats to a total alcohol dose of 6.6 g/kg/day, which was administered in two patterns: one group was exposed continuously (i.e., 24 hours per day) to alcohol for several days, resulting in an average peak BAC of 49 mg/dL. The second group received condensed exposure (i.e., only 12 hours per day) for the same number of days, resulting in an average peak BAC of 270 mg/dL. At the end of the treatment period, the brain weights of the rats in the condensed exposure group were significantly lower than those of the continuously exposed group, demonstrating that brain growth is impaired more by bingelike patterns of exposure to alcohol than by continuous exposure.

A subsequent study tested the hypothesis that a lower daily dose of alcohol can result in greater brain growth restriction and cell loss in various brain regions than will a higher daily dose if the lower dose is administered in a binge-like pattern (Bonthius and West 1990). The researchers compared the following three groups of alcohol-exposed neonatal rats:

- One group was exposed to 4.5 g/kg/day in a condensed pattern (i.e., for 4 hours per day); the resulting peak BACs averaged 365 mg/dL.
- A second group was exposed to the same dose administered in a less condensed pattern (i.e., for 8 hours per day), resulting in peak BACs averaging 195 mg/dL.
- A third group was exposed to a higher dose of alcohol (i.e., 6.6 g/kg/day) administered in a continuous pattern (i.e., for 24 hours per day); the resulting peak BACs averaged 45 mg/dL.

When the brains of all animals were weighed at the end of the study, the brain weights of the animals in the group receiving 4.5 g/kg/day over 4 hours were the lowest, followed by the brain weights of the animals receiving 4.5 g/kg/day over 8 hours (see figure). The animals receiving 6.6 g/kg/day administered continuously had the highest brain weights. These results clearly demonstrate that a lower daily dose consumed in a bingelike pattern can be more harmful than a higher daily dose consumed in a continuous pattern. The reason underlying these results is that the condensation of the alcohol administration into progressively shorter periods generated correspondingly higher peak BACs.

This relationship between pattern of alcohol exposure (and, consequently, peak BACs) and brain growth restrictions also has been observed in studies using nonhuman primates as experimental subjects. In an extensive and elegant series of experiments, researchers exposed nonhuman primates to alcohol in a bingelike pattern during different times of fetal brain development. The investigators administered alcohol to pregnant monkeys during gestation using 3 different administration periods: (1) the first 3 weeks, (2) the first 6 weeks, or (3) all 24 weeks of the gestation period (Astley et al. 1999). The monkeys received the alcohol in a binge-like pattern for 1 or 2 days per week (corresponding to a weekend binge), resulting in average peak BACs of 223 mg/dL. The study found that on several indices of gross brain development and cognitive functioning, no difference in the severity of the deficits existed among the infants of monkeys from the three timing groups. Thus, a similar amount of gross brain damage and impairment in cognitive function occurred if the fetuses were exposed to alcohol only during the early part of gestation, rather than throughout gestation.

Similar studies conducted on non-human primates demonstrated a variety of possible outcomes from alcohol exposure during early gestation. These outcomes included the loss of certain neurons (i.e., Purkinje cells) in the cerebellum^5^ (Bonthius et al. 1996), loss of certain neurons in the eye (i.e., retina ganglion cells) combined with various eye malformations (Clarren et al. 1990), and facial abnormalities similar to those seen in FAS children (Astley et al. 1999). Viewed together, these findings clearly demonstrate the vulnerability of the fetal brain to alcohol-induced damage from exposure during early gestation. Clinicians should emphasize these results, as well as those of the rat studies, when informing women of childbearing age about the harmful effects of binge-like alcohol exposure on fetal brain development during the first 4 to 6 weeks of pregnancy. These animal studies undeniably demonstrate that drinking cessation only after a pregnancy is realized may not spare the fetal brain and other organs from harm, including eventual behavior impairments.

## Results of Human Studies on the Effects of Maternal Drinking Patterns

Limited information exists on binge drinking and fetal outcome among humans in the clinical literature. One reason for this gap is the way that the information gathered from the human population is measured and categorized (e.g., see Gladstone et al. 1996). For example, measurements often focus on the average or total amount of alcohol consumed, rather than on drinking patterns or the maximum alcohol dose consumed on a single occasion. For example, as mentioned earlier, binge drinkers may consume lower total alcohol amounts than do continuous drinkers, because in actuality, binge drinkers consume fewer drinks. Thus, if consumption is averaged over time (e.g., 1 week or 1 month), a binge drinker’s consumption may be classified as light or moderate, suggesting little risk. Furthermore, some alcohol-intake questionnaires inquire about the typical alcohol intake per occasion, rather than the maximum alcohol intake on a single occasion. Averaging the typical amount of alcohol consumed per occasion during a period of 1 month or 1 year may seriously underestimate the maximum amount of alcohol consumed in a single episode of binge drinking. Averaging may lead to particularly ambiguous results, because some animal studies have reported neuronal loss in the developing brain even after only a single episode of binge exposure (Goodlett and Eilers 1997; Pauli et al. 1995).

Nevertheless, several studies have assessed the effects of maternal drinking patterns on the outcome of human offspring. Some of those studies have failed to find a relationship between binge alcohol consumption and adverse offspring outcome; however, such studies are typically plagued with methodological problems (e.g., see Gladstone et al. 1996).

One detailed series of studies examining the effects of binge exposure on offspring development has been conducted by researchers in the Seattle Longitudinal Prospective Study on Alcohol and Pregnancy under the direction of Dr. Ann Streissguth. In this study, pregnant women were asked to estimate their alcohol consumption in terms of quantity (i.e., how much alcohol they drank), frequency (i.e., how often they drank alcohol), and variability (i.e., when they drank alcohol relative to the stages of pregnancy) for two time periods—at midpregnancy and during the month prior to pregnancy recognition (Streissguth et al. 1989). This approach enabled the researchers to calculate scores that reflected both the level and pattern of alcohol consumption. Then, the children of those women were followed for at least 14 years and were assessed at specific ages on several different tasks that reflected learning, memory, and various forms of cognitive processing.

When the children were studied at age 7.5, maternal binge drinking (i.e., consumption of five or more drinks per occasion) in the month prior to pregnancy recognition was the best predictor of neurobehavioral deficits in attention, memory, and cognitive processing as well as inflexibility in problem-solving. Moreover, the children exposed to binge drinking before their mothers realized that they were pregnant were more likely to be rated by their parents as having learning problems, being below average academically, and being hyperactive and impulsive. The children also were more likely to be rated by teachers as expressing behaviors that were incompatible with learning (Streissguth et al. 1990). It is particularly important to note that the commonly used measure of alcohol consumption—average ounces of alcohol consumed per day and frequency of drinking—did not predict these neurobehavioral outcomes.

When the children were studied again at age 11, children of binge-drinking mothers were still classified as having problems with distractibility, restlessness, and lack of persistence (Olson et al. 1992). Finally, when the children reached age 14, the variable “number of drinks per occasion” still significantly predicted some critical neurobehavioral measures (i.e., problems with response inhibition and fluctuating attention span) (Streissguth et al. 1994*b*). Thus, one common theme emerged from this comprehensive data analysis—the greater the average number of drinks that the pregnant women consumed per occasion, the worse her children later performed on measures important for reading and arithmetic skills (Streissguth et al. 1994*a*).

The harmful effects of bingelike drinking patterns were confirmed with more recent data from a sample of mothers and their infants studied in Detroit, Michigan (Jacobson et al. 1998). The investigators found that infants born to mothers who consumed at least five drinks per occasion and, on average, at least once per week, had clearly definable functional deficits as measured by birth weight, the Bayley Mental and Psychomotor Developmental scales, the Elicited Play test, and tests of processing speed. The researchers calculated that the threshold for these effects was an average consumption of 0.50 ounces of absolute alcohol per day when the alcohol was consumed in a bingelike fashion. The functional deficits were not observed, however, in the offspring of women who reported drinking frequently but not in a bingelike pattern.

## Why is Binge Drinking More Harmful Than Other Drinking Patterns?

Several factors may help explain why binge drinking is particularly harmful to fetal brain development. First, the peak BACs achieved with this drinking pattern are higher than the peak BACs achieved with more continuous drinking patterns. In turn, a high BAC is a critical factor in producing fetal brain injury. Researchers do not yet know the minimum amount of alcohol that is harmful to the developing brain. It is possible that the fetus can tolerate a certain level of alcohol exposure with no ill effects, and that only by binge drinking, a woman would risk exceeding such a threshold for alcohol-induced fetal brain injury. Another consequence of higher peak BACs is prolonged alcohol exposure. The rate of alcohol breakdown (i.e., metabolism) in the body remains the same regardless of the amount of alcohol consumed. As a result, the higher the BAC is, the longer it takes the body to metabolize all the alcohol. Accordingly, binge-drinking episodes produce longer periods of alcohol exposure for the developing fetal brain than do consumption and metabolism of a single drink.

Second, the timing of binge-drinking episodes relative to key stages of brain development may influence the extent of its adverse effects. Binge drinking may be particularly harmful if it occurs at a point in development that is critical for some specific developmental brain event. Because some of these developmental events are of short duration, a single binge-drinking episode might overlap with one of these events. Unfortunately, the prevalence of bingelike alcohol consumption is particularly high among women (and men) of reproductive age; moreover, researchers have found a strong association between binge drinking and unplanned or unprotected sexual activity (Wechsler et al. 1994; Parker et al. 1994). Consequently, binge drinking may increase episodes of unprotected sexual intercourse and, thus, increase the risk of alcohol exposure near conception.

Third, alcohol consumption in a bingelike pattern exposes the developing fetal brain to not only high peak BACs, but also to periods of withdrawal from alcohol. The effects of multiple withdrawal episodes may be a potentially important risk factor for developmental brain injury. To date, no comprehensive studies have examined in detail the effects of oral, bingelike alcohol exposure and the resulting withdrawal during key stages of fetal brain development. Some of the deficits in brain function observed in affected children, however, may be related to severe physiological responses to the withdrawal from alcohol as well as to the exposure to alcohol. This hypothesis is an underrepresented and important area of research that requires further attention.

## Conclusions

The evidence presented in this article allows for two critical conclusions. First, the data from the comprehensive series of studies performed on humans, as well as on nonhuman primate and rodent experimental models, clearly show that binge drinking is more harmful to fetal brain development than is non-binge drinking. Second, early alcohol exposure (i.e., during the first few weeks of gestation in humans) may have as much adverse effect on fetal brain development as would alcohol exposure throughout pregnancy. Based on these findings, one might argue that women who engage in binge drinking during the early part of their pregnancy could continue to drink without further consequence. This assumption is incorrect, however, because some aspect of development or cognition that is not measured in the cited studies would most likely be impaired to a greater extent by continuous alcohol exposure throughout pregnancy than by limited exposure during early pregnancy.

Because no threshold for alcohol’s detrimental effects on the developing fetal brain has been established, women should cease to consume alcohol immediately upon learning that they are pregnant. It would be best (i.e., safest), however, that women who are contemplating pregnancy consider changing their drinking habits even before conception. Accordingly, appropriate educational efforts should be directed especially toward young women, who may engage in binge-drinking patterns that can lead to unprotected sexual intercourse and unplanned pregnancies (although men may be held equally accountable for the prevention of unplanned pregnancies). Fortunately, most women reduce or stop their alcohol consumption as soon as they realize that they are pregnant; in some cases, however, some injury to the fetal brain may have already occurred. Even if a mother drank heavily during parts of her pregnancy, abstinence during the remainder of the pregnancy is likely to have beneficial effects on the developing fetus.

## Figures and Tables

**Figure:**
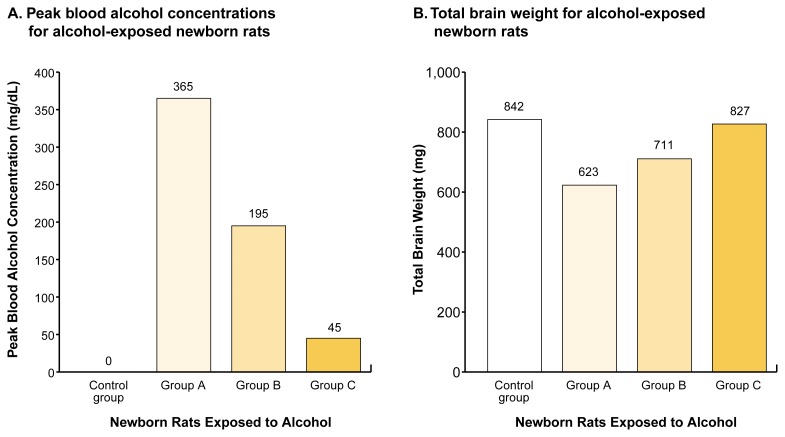
Effects of different patterns of neonatal alcohol exposure on brain development in rats. Newborn rats received alcohol according to three different schedules from day 4 to day 10 after birth. Group A received a total of 4.5 grams of alcohol per kilogram of bodyweight per day (g/kg/day), administered over 4 hours each day. Group B received the same amount of alcohol (4.5 g/kg/day), but it was administered over 8 hours each day. Group C received a total of 6.6 g/kg/day, administered over 24 hours each day. Control animals received no alcohol during that time. The peak blood alcohol concentrations (BACs) for each group are shown in panel A. On day 10, the animals’ total brain weights were measured (panel B). Group A showed the lowest brain weights, whereas the brain weights of group C were almost normal. The results suggest that higher peaks in BAC are associated with greater reductions in brain weight.

## Footnotes

1 The corpus callosum is a bundle of nerve fibers that connects the right and left hemispheres of the brain.

2 People with a BAC of 0.08 percent to 0.10 percent are considered legally intoxicated.

3 The term “artificial rearing methods” means that newborn rats are raised away from their mothers, in climate- and humidity-controlled environments, and fed a nutritionally adequate diet similar to that found in rat mothers’ milk. Alcohol can be added to that diet to generate groups of rat pups exposed to various alcohol doses and exposure patterns

4 On average, and if all variables are experimentally controlled, a 120-lb woman may achieve the range of BACs discussed in this article if she consumes approximately the following number of standard drinks in a binge-like manner: 49 mg/dL = 1.5 drinks; 195 mg/dL = 6.25 drinks; 223 mg/dL = 7.5 drinks; 270 mg/dL = 8.75 drinks; 365 mg/dL = 11.5 drinks; 525 mg/dL = 15+ drinks.

5 The cerebellum is a region located at the base of the brain that is involved in motor and cognitive functions.
